# Predicting pathological complete response by comparing MRI‐based radiomics pre‐ and postneoadjuvant radiotherapy for locally advanced rectal cancer

**DOI:** 10.1002/cam4.2636

**Published:** 2019-10-22

**Authors:** Yuqiang Li, Wenxue Liu, Qian Pei, Lilan Zhao, Cenap Güngör, Hong Zhu, Xiangping Song, Chenglong Li, Zhongyi Zhou, Yang Xu, Dan Wang, Fengbo Tan, Pei Yang, Haiping Pei

**Affiliations:** ^1^ Department of Gastrointestinal Surgery Xiangya Hospital Central South University Changsha China; ^2^ Department of General Visceral and Thoracic Surgery University Medical Center Hamburg‐Eppendorf Hamburg Germany; ^3^ Department of Rheumatology Guangdong Provincial People's Hospital Guangdong Academy of Medical Sciences Guangzhou China; ^4^ Department of Cardiology Xiangya Hospital Central South University Changsha China; ^5^ Department of Thoracic surgery Fujian Provincial Hospital Fuzhou China; ^6^ Department of Oncology Xiangya Hospital Central South University Changsha China; ^7^ Department of Oncology Hunan Cancer Hospital Changsha China

**Keywords:** locally advanced rectal cancer, MRI‐based radiomics, neoadjuvant chemoradiotherapy, pathologic complete response, predictive model

## Abstract

**Background:**

Total mesorectal excision following neoadjuvant chemoradiotherapy (nCRT) is recommended in the latest treatment of locally advanced rectal cancer (LARC).

**Objective:**

To predict whether patients with LARC can achieve pathologic complete response (pCR), comparing MRI‐based radiomics between before and after neoadjuvant radiotherapy (nRT) was performed.

**Methods:**

One hundred and sixty‐five MRI‐based radiomics features in axial T2‐weighted images were obtained quantitatively from Imaging Biomarker Explorer Software. The specific features of conventional and developing radiomics were selected with the analysis of least absolute shrinkage and selection operator logistic regression, of which the predictive performance was analyzed with receiver operating curve and calibration curve, and applied to an independent cohort.

**Results:**

One hundred and thirty‐one target patients were enrolled in the present study. A radiomics signature founded on seven radiomics features was generated in the primary cohort. A remarkable difference about Rad‐score between pCR and non‐pCR group occurred in both of primary (*P* < .001) or validation cohorts (*P* < .001). The value of area under the curves was 0.92 (95% CI, 0.86‐0.99) and 0.87 (95% CI, 0.74‐1.00) in the primary and validation cohorts, respectively. The Rad‐score (OR = 23.581; *P* < .001) from multivariate logistic regression analysis was significant as an independent factor of pCR.

**Conclusion:**

Our predictive model based on radiomics features was an independent predictor for pCR in LARC and could be a candidate in clinical practice.

## Graphical abstract

This study innovatively compared variation on MRI‐based features in the process of concurrent chemoradiotherapy. Our study about a predictive model with radiomic features was promising in the prediction of pCR to neoadjuvant chemoradiation in LARC. Our method developed with information from the clinical obtained T2‐weighted sequence may be pragmatic as a complement in clinical strategy making.

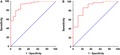

## 1 INTRODUCTION

Colorectal cancer (CRC) is considered as the third top malignancy in the world,^1^ approximate 30%‐50% of which is rectal cancer.^2^ Currently, the recommended treatment for locally advanced rectal cancer (LARC, T3‐4 or N+) is total mesorectal excision (TME) after neoadjuvant chemoradiotherapy (nCRT).^3^ And neoadjuvant radiotherapy (nRT) plays an important role in nCRT. However, different patients bring the wide variabilities out of the response of LARC to nCRT, with a ladder from no tumor regression to pathologic complete response (pCR).^4^ Although the necessity of surgery in LARC patients with pCR is a subject of ongoing argument, the majority of patients are still undergoing surgery in practice. Considering surgical complications, especially after nCRT, and outstanding long‐term outcomes in pCR patients apart from surgery, Habr‐Gama et al proposed the “watch‐and‐wait” approach first.^5^ Thus, the identification of pCR before surgery gains more and more concerns in therapeutic management.

Radiomics, which extracted excavatable high‐dimensional data from digital images, revealed nonvisual information associated closely with underlying pathophysiology and even tumor heterogeneity.^6^, ^7^ Recently, the development of radiomics has shown great potential for therapy guidance and tumor prognosis across various types of cancer.^8^, ^9^, ^10^, ^11^

Despite of diverse outcomes, several researches displayed the potential significance of imaging modalities.^12^, ^13^, ^14^, ^15^, ^16^ Among all modalities, magnetic resonance imaging (MRI) was regarded as the most recommended and promising method because it showed high soft tissue resolution without radiation to damage human body, and had a wide routine clinical application for the evaluation of rectal cancer. Several predicting models also based tumor response to nCRT on MRI‐related radiomics in LARC. However, all of the studies only focused on the MR images prior to nCRT, which might have inherent limitations to reflect the impact of nCRT on target population.

Therefore, we were planning to investigate whether the difference of quantitative MRI‐based radiomics analysis between pre‐nRT and post‐nRT can be of great help to predict pCR in LARC.

## 2 MATERIALS AND METHODS

### 2.1 Patients

**Figure 1:**
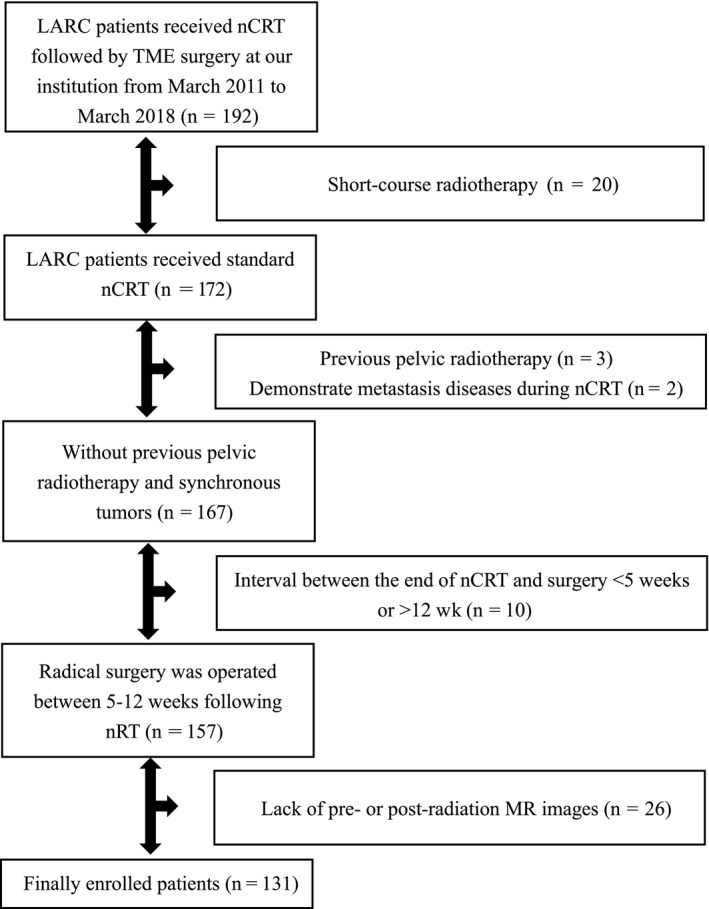
Flowchart

This study collected the medical information of consecutive patients with LARC, who treated with nCRT followed by radical surgery (total mesorectal excision) between March 2011 and March 2018 in Xiangya hospital. Biopsy‐proven rectal adenocarcinoma was performed before receiving radiotherapy and/or chemotherapy for patients. Locally advanced rectal cancer was defined as T3‐4 or N+ (c‐Stage II‐III) without any evidence of distant metastases in clinical stage, and evaluated by pelvic magnetic resonance imaging (MRI), chest X‐ray, digital rectal examination, abdomen, pelvis and/or chest contrast‐enhanced computed tomography (CT), endorectal ultrasonography (ERUS), and/or bone single‐photon emission computed tomography (SPECT). Exclusion criteria contained short‐course radiotherapy only, synchronous tumors, lack of pre‐ or postradiation MR images, interval between the end of nRT and surgery <5 weeks or >12 weeks, and previous pelvic radiotherapy (Figure 1).

### 2.2 Protocol of image acquisition and extraction of radiomic features

MR images were acquired with a 1.5‐T superconductive unit (MAGNETOM Sonata, Siemens, Erlangen, Germany; Singa HDxt, GE Medical Systems, Umatilla, FL, USA). Coronal, sagittal turbo spin‐echo T2‐weighted images, and transverse T1/T2‐weighted images were included in the sequences. The pre‐nRT MRI was obtained within 2 weeks before nRT and post‐nRT MRI was gained within 1 week after nRT.

One hundred and sixty‐five MRI‐based radiomics features (Supplementary material S1), which can quantify tumor's volume, intensity, and texture property, were extracted from manual segmentation, including pre‐ and post‐nRT MR images, by imaging biomarker explorer software (IBEX). The regions of interest (ROI) were outlined along the edge of tumor, and it took approximately 5 min to proceed segmentation manually for each tumor. Segmentations of ROI were operated manually by Y.P—a radiotherapist with 10 years of experience in rectal MR imaging and reaffirmed by H.Z—a radiologist with 20 years of experience. The two radiotherapists were both blinded to the clinical data. Radiomic features for each of included patient were automatically calculated by the software following tumor segmentation.

The data we used for statistical analysis were obtained by subtracting quantitative MRI‐based radiomic features of post‐nRT from that of pre‐nRT.

### 2.3 Treatment

The 6‐week administration of neoadjuvant radiotherapy was at a dose of 46‐50 Gy in 23‐25 fractions (2 Gy/fraction, 5 d/wk) for the whole pelvis, and 6‐8Gy in 3‐4 fractions for the primary tumor. Radiotherapy machine included Trilogy, 23EX, D‐2100CD (Varian) and TomoTherapy H^TM^ Series 2.1.x Hi Art 5.1x (Accuray Incorporated). All patients had a CT emulation of three‐dimensional conformal planning and intensity‐modulated radiotherapy (IMRT), concomitant with a three‐field treatment plan involving a 6‐MV photon posterior‐anterior field and 15‐MV photon opposed lateral fields.

All patients received the treatments of neoadjuvant concurrent chemotherapy based on 5‐fluorouracil (5‐FU). Either of the two programs was selected: mono‐chemotherapy of 5‐FU: bolus injection [400 mg/m^2^/d] for continuous 5 days in the first and last weeks of radiotherapy or oral capecitabine [825 mg/m^2^] twice per day during weekend breaks of radiotherapy; combined chemotherapy: mFOLFOX6 (bolus infusion of 5‐FU [400 mg/m^2^] 2 hours on d1, continuous intravenous drip of 5‐FU [1200 mg/m^2^/d] 46 hours on d1‐2, intravenous drip of leucovorin [400mg/m^2^] 2 hours on d1, intravenous drip of oxaliplatin [85 mg/m^2^] 2 hours on d1, 2 weeks per cycle) or CAPOX (oral capecitabine [1000 mg/m^2^] twice daily d1‐14, intravenous drip of oxaliplatin [130 mg/m^2^] 2 hours on d1, 3 weeks per cycle).

TME surgery was operated between 5 and 12 weeks following nRT, and the surgical strategy, including abdominoperineal resection (APR), trans‐anal resection (TAR), low anterior resection (LAR), and LAR plus prophylactic ileostomy, was made by surgeon.

### 2.4 Tumor response evaluation

The tumor tissue was sampled prior to paraffin embedding and slicing into 4‐mm‐thick sections to evaluate the tumor response to nCRT after resection. pCR, no viable tumor cells in the bowel wall (T stage) and regional nodes (N stage)‐‐ypT0N0, was equivalent to the tumor regression grade (TRG) 0,^17^ which is fibrotic mass, acellular mucin pools or hyaline degeneration only, without detecting tumor cells (complete regression). The other pathological conditions, including TRG 1‐4^17^ (no regression, minimal regression, moderate regression, and near‐complete regression), were defined as non‐pCR.

### 2.5 Data collection

The parameters were appraised as latent clinical predictors of tumor response to nCRT as follows: age, gender, Body Mass Index (BMI), clinical T (cT) stage, clinical lymph node (cN) status, distance from the anal verge, histologic type, pre‐nCRT CEA level, concurrent chemotherapy regimen and interval time between nCRT and surgery.

Clinical T classification was judged by pelvic MRI and/or ERUS. Smallest diameter of a regional lymph node ≥5 mm observed on pelvic MRI was defined as positive lymph node involvement.^18^ The distance between the tumor and the anal verge was measured by MRI. The clinical TNM staging was originated from the 8th edition of the American Joint Committee on Cancer (AJCC) Staging system. The peripheral blood within 2 weeks prior to nCRT under the condition of abrosia was extracted for the examination of pre‐nCRT serum tumor markers levels.

### 2.6 Construction of Rad‐score with the LASSO regression model

Since the multicollinearity among radiomics features existed, the optimal subset of radiomic features was selected by the LASSO binary logistic regression model in order to establish the radiomic signature score (Rad‐score). And a penalty parameter (also called as tuning parameter) was brought into the mechanism of the LASSO regression to penalize the coefficient of variables embodying in the LASSO regression model, averting the issue of overfitting. With the raise of tuning parameter (λ), more coefficients were installed to zero (less variables were chosen), and more shrinkage was applied among the nonzero coefficients. The region under the receiver operating characteristic curve was constructed vs log(*λ*) to find out the optimal value of log(λ) with the minimum criterion and the one standard error of the minimum criterion. LASSO binary logistic regression analysis was performed in the Bglmnet^ package of R software, and the process of programming is presented in the Supplementary material S1.^19^, ^20^, ^21^

### 2.7 Statistics

Intergroup comparisons were analyzed using Pearson's chi‐square test, Mann‐Whitney *U* test, Fisher's exact test, or Student's *t* test, according to the nature of the data. The independent prognostic factors were selected by multivariable logistic regression analysis. The performance of the model was evaluated in the primary and validation cohorts. The discrimination of the signature was evaluated through the area under the curve (AUC). The apparent calibration curve was drawn with model‐predicted probability vs actual probability of invasive adenocarcinoma, and the bias‐corrected curve was generated from 1000 bootstrap resamples. SPSS from Windows, version 20.0 (IBM) was used for statistical analysis. A difference was considered significant at *P* < .05 with two sides.

## 3 RESULTS

### 3.1 Patients characteristics

One hundred and thirty‐one target patients were contained in our study. The parameter of patients in the primary and validation cohorts was listed in Table 1. Patients were randomly distributed into primary cohort and validation cohort in the ratio of 2:1 to build the pCR predictive model. In the primary cohort, 63.22% of target population was male, whose age was 51.18 years in average. In the validation cohort, more than half of patients were male (59.09%) with an average age of 51.64.

**Table 1:** Characteristics of patients in the primary and validation cohorts

Characteristic	Primary cohort(n = 87)			Validation cohort(n = 44)		
	non‐pCR	pCR	*P*	non‐pCR	pCR	*P*
Gender			.837			.614
Male	44 (63.77%)	11 (61.11%)		20 (57.14%)	6 (66.67%)	
Female	25 (36.23%)	7 (38.89%)		15 (42.86%)	3 (33.33%)	
Age (y)	51.35 ± 11.49	50.56 ± 10.31	.791	50.49 ± 11.14	56.11 ± 9.49	.173
BMI (kg/m^2^)	22.69 ± 3.18	22.19 ± 2.75	.546	21.90 ± 2.92	23.20 ± 2.68	.234
Distance from the anal verge (mm)	40.97 ± 14.33	35.82 ± 9.99	.155	38.49 ± 14.65	39.88 ± 13.13	.797
Pathology type			.989			.210
Well/moderately differentiated	49 (71.01%)	12 (66.67%)		29 (82.86%)	9 (100%)	
Poor differentiated	13 (18.84%)	5 (27.78%)		2 (5.71%)	0 (0.00%)	
Mucinous carcinomas	7 (10.15%)	1 (5.55%)		4 (11.43%)	0 (0.00%)	
Clinical T staging			.508			.090
cT2	8 (11.59%)	3 (16.67%)		3 (8.57%)	1 (11.11%)	
cT3	46 (66.67%)	12 (66.66%)		20 (57.14%)	7 (77.78%)	
cT4	15 (21.74%)	3 (16.67%)		12 (34.29%)	1 (11.11%)	
Clinical N staging			.740			.732
cN0	18 (26.09%)	4 (22.22%)		6 (17.14%)	2 (22.22%)	
cN1	14 (20.29%)	3 (16.67%)		7 (20.00%)	1 (11.11%)	
cN2	37 (53.62%)	11 (61.11%)		22 (62.86%)	6 (66.67%)	
pre‐CEA (ng/mL)	3.49 (1.42‐11.85)	4.75 (1.92‐6.14)	.608	6.77 (2.71‐15.46)	0.98 (0.76‐1.89)	.000
Chemotherapy regimen			.006			.548
Mono‐chemotherapy	61 (88.41%)	11 (61.11%)		28 (80.00%)	8 (88.89%)	
Combined chemotherapy	8 (11.59%)	7 (38.89%)		7 (20.00%)	1 (11.11%)	
Interval to surgery (wk)	7 (6‐9.25)	8 (6‐11)	.101	9 (5.5‐11.5)	9 (7‐10)	.988
Rad‐score	−1.74（−2.16 to −1.40）	−0.57 (−1.01 to 0.10)	<.001	−1.77 (−2.20 to −1.21)	−0.55 (−1.23 to −0.10)	<.001

The percentages of patients with pCR in the primary cohort and the validation cohort were 20.69% (18/87) and 20.45% (9/44), respectively. Chemotherapy regimen was significantly different between the pCR and non‐pCR groups for the primary (*P* = .006) but not validation (*P* = .548) cohorts. Conversely, there was a conspicuous difference of pre‐CEA level between the pCR and non‐pCR groups in the validation (*P* < .001) but not primary (*P* = .608) cohorts. The difference about clinical T staging, clinical N staging and the interval weeks between CRT and surgery were not observed in both of the primary or validation cohort.

### 3.2 Feature selection of the radiomic signature

Aggregate 165 features were obtained from T2‐weighted images for individuals (both pre‐nRT and post‐nRT) by IBEX software. In order to incarnate the variations on 165 MRI‐based features in the process of concurrent chemoradiotherapy, the analytical data were obtained by subtracting quantitative features of post‐nRT from that of pre‐nRT. A set of features with corresponding numbers were selected by LASSO and used to calculate the Rad‐scores for the pCR model.

**Figure 2:**
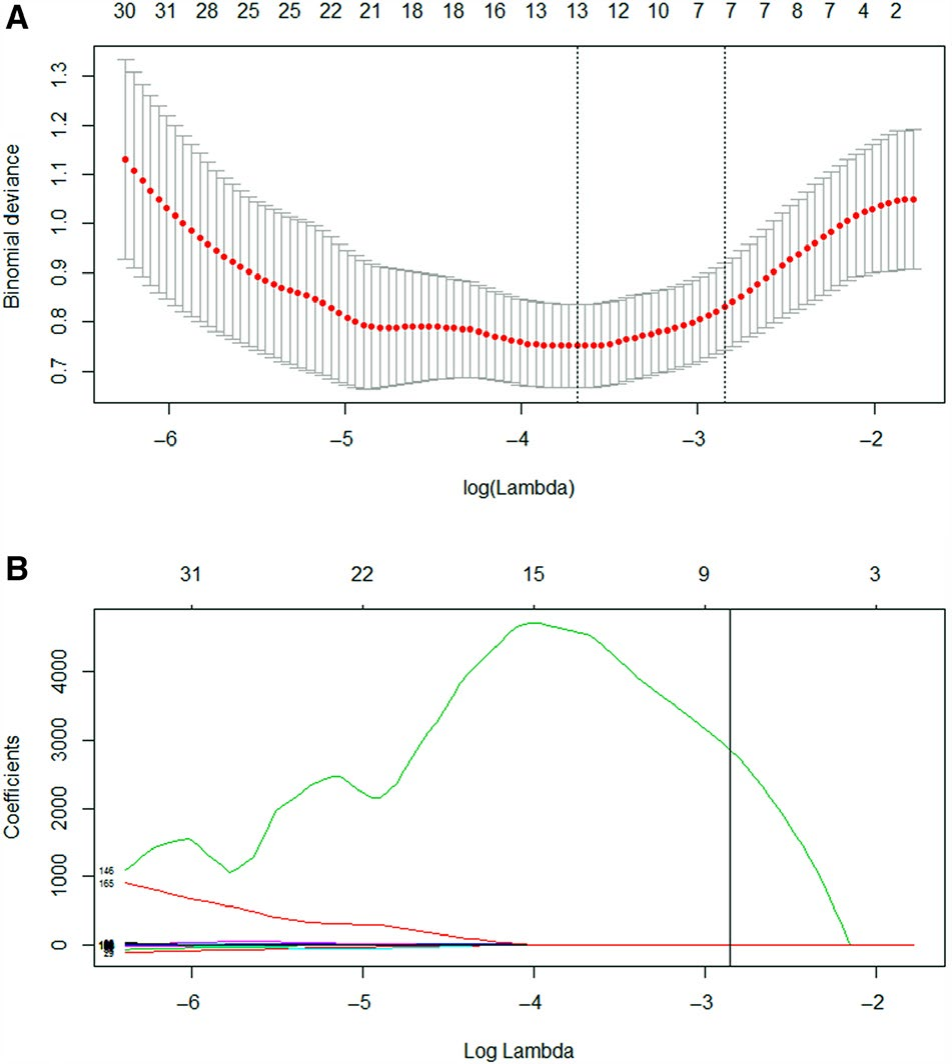
Radiomic feature selection using LASSO regression model. A, Optimal feature selection according to AUC value; (B) LASSO coefficient profiles of the 165 radiomic features. Vertical line was drawn at the selected value using 10‐fold cross‐validation, where optimal λ resulted in 7 nonzero coefficients

*λ* was chosen by 10‐fold cross‐validation in the LASSO model, and log(*λ*) of −2.85 was the optimal subset for seven radiomics features, at which these potential predictors, including GOH‐Skewness, GLRLM‐Run Length Non‐uniformity, ID‐Local Entropy Max, ID‐ Local Range Min, NIDM‐Coarseness, maximum 3D diameter, and Surface Area Density, were extracted from 165 radiomic features with nonzero coefficients of the LASSO logistic regression model for the primary cohort (Figure 2). Both Figure 1A,B showed that the number of variables contained into the model was decreased, and the absolute values of the coefficients for the variables also sank toward zero as log(λ) altered from 6 to 0.

**Figure 3:**
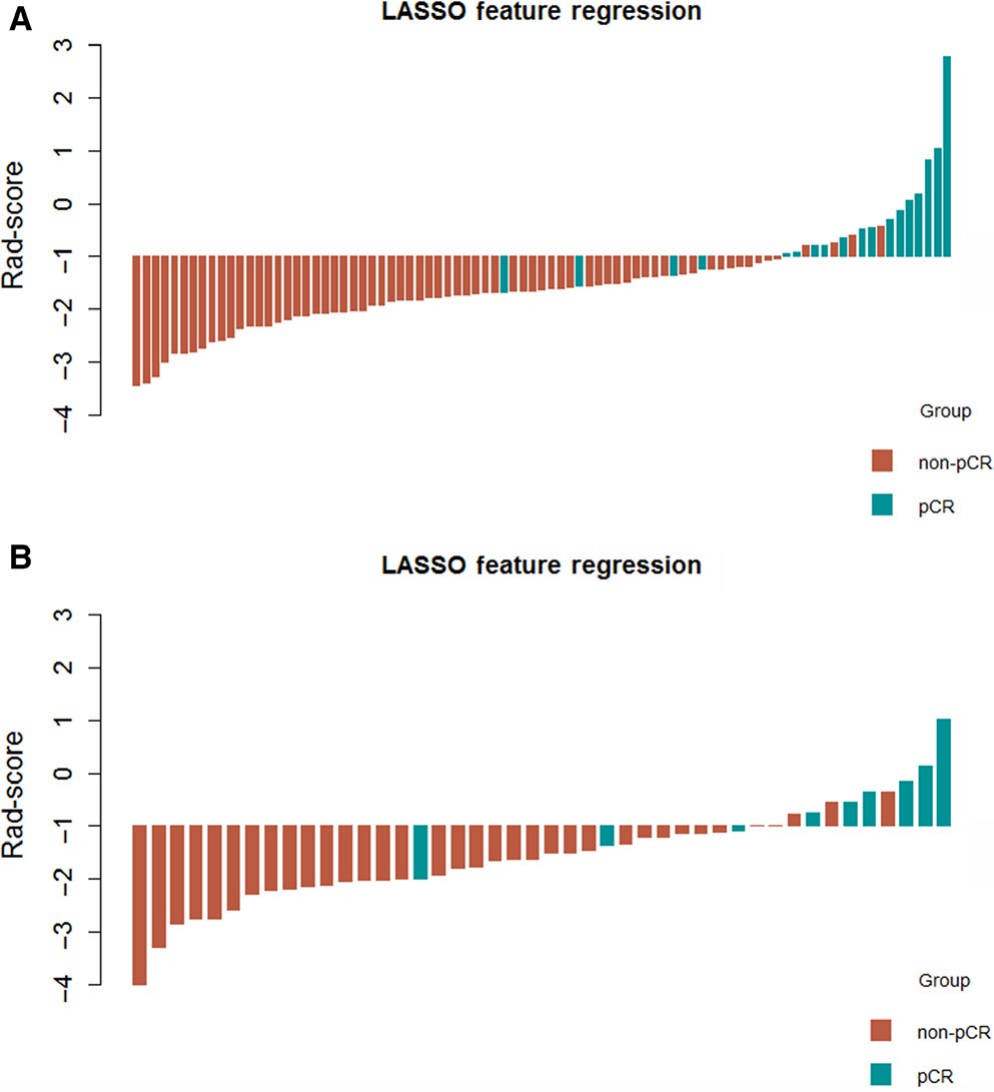
Rad‐score for patients in (A) the primary cohort and (B) the validation cohort

The radiomic signature score (Rad‐score) was assessed for each patient founded on the seven radiomic features (Supplementary material S1). Waterfall plots showed the Rad‐score for individuals in primary (Figure 3A) and validation cohort (Figure 3B). There was a marked difference of Rad‐score between pCR and non‐pCR group regardless of the primary (*P* < .001) or validation cohort (*P* < .001). pCR was associated with higher mean value of Rad‐score in both the primary and validation cohort (−0.57 and −0.55, respectively) compared to non‐pCR group (–1.74 and –1.77, respectively).

### 3.3 Performance of the radiomics signature

**Figure 4:**
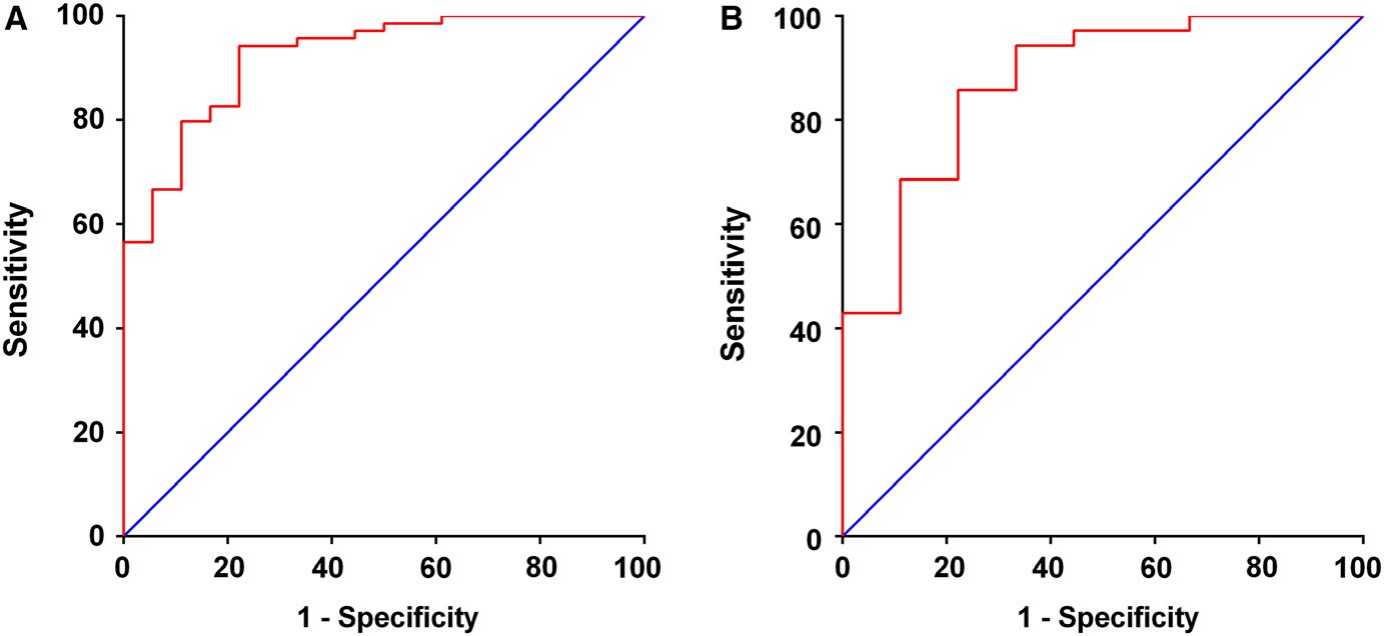
Area under the curve (AUC) of MRI‐based radiomics model in (A) the primary cohort and (B) the validation cohort

**Figure 5:**
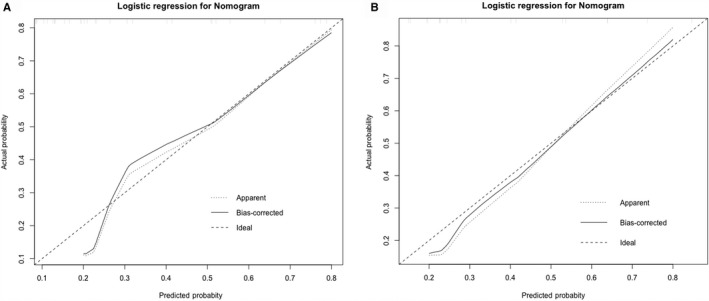
Calibration curve showing the predicted vs actual probability for pCR. Calibration curve of radiomics signature in (A) the primary cohort and (B) the validation cohort

Variables with differences (*P* < .2) in univariate analysis were selected into the Logistic regression model of multivariate analysis. Rad‐score (OR = 23.581; *P* < .001) was identified as independent factors in multivariate logistic regression analysis (Table 2). The value of AUCs was 0.92 (95% CI, 0.86‐ 0.99) in the primary cohort and 0.87 (95% CI, 0.74‐1.00) (Figure 4) in the validation cohort. The calibration curve of the signature was presented in Figure 5, indicating that the model made accurate predictions.

**Table 2:** Results of multivariate logistic regression analysis

Characteristic	*β*	Odds ratio (95% CI)	*P*
Intercept	4.861		
Distance from the anal verge (mm)	−0.041	0.959 (0.900‐1.023)	.205
Chemotherapy regimen	0.808	2.244 (0.320‐15.749)	.416
Interval to surgery (wk)	−0.121	0.886 (0.621‐1.624)	.504
Rad‐score	3.160	23.581 (4.445‐125.090)	<.001

Abbreviations: *β*, regression coefficient; CI, confidence interval.

## 4 DISCUSSION

Increasing data supported that pCR following nCRT in LARC was linked to prominent enhanced local control, reduced incidence of distant metastasis, and long‐term survival compared with non‐pCR.^22^ With the excellent advantage, it had been prompting nonoperative managements, including a “watch‐and‐wait” proposal, in selected LARC patients.^5^ However, the pCR rate was unsatisfactorily low, hovering at around 20% (range 15%‐27%).^22^ Our pCR incidence (20.61%) was also within the range. Hence, identifying the predictive factors of pCR played a key role while attempting to improve the pCR, especially in term of averting more invasive treatments. Due to the superiority of effective availability and broad applicability, clinical characteristics were broadly discussed. Although there were few researches showing that clinical characteristics affect pCR after nCRT, some potent clinical predictive factors, especially radiomics, emerges and gain more attentions. Numerous studies indicated that radiomic model can evaluate tumor heterogeneity, and correlate radiological findings with underlying genomic and biological characteristics, including treatment response and prognosis.^6^, ^23^ Moreover, the large amount of previous evidences supported the application of advanced MRI‐based radiomic features for predict of tumor responses to nCRT in LARC patients.^24^, ^25^

Our study was in general consistent with prior researches. Nie^26^ and Cui^27^ reported a relatively satisfactory result by using a radiomics method, with AUCs of 0.84 and 0.94 for pCR prediction, respectively. However, there was an obvious predominance in our study compared to their studies. First, we innovatively compared variation on MRI‐based features in the process of concurrent chemoradiotherapy, which was a promising guidance in the tumor change and treatment response. All other studies only analyzed preradiotherapy MRI images but ignored postradiotherapy. In fact, the development of functional MRI sequences has enabled us to assess tumor characteristics of post‐nCRT MRI.^28^ A large prospective trial in the MRI and Rectal Cancer European Equivalence (MERCURY) study revealed that standard morphological MRI (*T*_2_ weighted) had a close association with survival outcomes,^29^ indicating the important role of post‐nRT MRI assessment of tumor regression grade in prognosis. Second, our radiomic features were acquired from only one sequence, such as the T2‐weighted images. The T2‐weighted images are commonly used in clinical practice, which is familiar to radiologists. In addition, T2‐weighted images are quite stable and can be acquired easily. In contrast, diffusion‐weighted images (DWI) are prone to distortion and susceptibility artifacts, causing the inaccuracy of tumor segmentation and data extraction. Similarly, other sequences including T1‐weighted dynamic contrast enhanced images depend on the amount and distribution of the injected contrast‐enhancing agent, which might be influenced by the variable hemodynamic conditions in the patients.

It was already clear that CRC is a heterogeneous disease, and tumor spatial heterogeneity is a critical predictor for prognosis. Image texture analysis is a feasible approach of quantifying heterogeneity.^30^ Our study suggested that a creative radiomic signature founded on seven radiomic features was an independent predictor for pCR in LARC after nCRT. Among these seven features, GOH‐Skewness, ID‐Local Entropy Max, ID‐ Local Range Min, GLRLM‐Run Length Non‐uniformity, and NIDM‐Coarseness associated with the heterogeneity of tumor.^23^, ^30^ GOH‐Skewness, ID‐Local Entropy Max, and ID‐Local Range Min were gained from various histograms of voxel intensities. NIDM‐Coarseness is the level of alterations in the intensity of spatial rate. GLRLM‐Run Length Non‐uniformity assesses the distribution of runs over the run lengths. Radiomics can have objective reflections on both the attenuation and dispersion of gray level intensity through quantitative analysis for MR images, which may be less apparent in direct visual assessment.^31^ Although the best way to determine tumor heterogeneity is to detect molecular subtypes using tissue specimens, which taken by colonoscopy are only sufficient for pathological diagnosis. Therefore, MRI‐based radiomics analysis helps us to deepen the understanding of CRC disease, improve the diagnosis, and assessment therapy response after nCRT.

As a conventional diagnostic performance, diminutive tumor size was associated with pCR in several studies.^32^ Our previous research also reached the same conclusion.^33^ However, the value of AUC for tumor size was not ideal^33^—only 0.629 in the previous study. In this study, our radiomics model contained not only an indicator of tumor size—Max 3D Diameter, but also a tumor density indicator—Surface Area Density, whose variation might not be evident on direct visual assessment. Therefore, we believe that our predictive model can improve the accuracy of prediction and ameliorate the applicability in clinic.

Interestingly, our study demonstrated that there was a marked difference about chemotherapy regimen between the pCR and non‐pCR groups in the primary (*P* = .006) but not validation (*P* = .548) cohorts. Meanwhile, there was no difference in multivariate logistic regression analysis between combined chemotherapy regimens and mono‐chemotherapy in primary cohort (*P* = .416). Therefore, this study believed that the advantage of combined chemotherapy regimen requires further clinical studies to confirm, concerning that only a few studies indicated a higher pCR under the condition of another agent added to 5‐FU‐based nCRT.^34^ Conversely, pCR was associated with pre‐CEA level compared with non‐pCR groups in the validation (*P* < .001) but not primary (*P* = .608) cohorts. Another study suggested that pre‐nCRT CEA levels could be a predictor for prognosis of local tumor control but not for pCR.^35^ In fact, both of chemotherapy regimen and pre‐nCRT CEA were meaningless in multivariate analysis.

This predictive model in our study can report the sensitivity of neoadjuvant chemoradiation better, which was closely related to survival.^33^ Moreover, the predictive model can provide more reliable information on whether patients can achieve pCR, which can be a firm support for patients to perform “watch‐and‐wait” proposal. Retrospective data with the limited number of patients from single institution may affect the reliability to some extent in our study. Consequently, more prospective randomized trials from various regions are exactly needed to get a better comprehension in promoting the individualized nCRT for LARC.

In conclusion, our study showed a predictive model with radiomic features was promising to predict pCR to neoadjuvant chemoradiation in LARC patients. In addition, our method developing with information from the clinical obtained T2‐weighted sequence may be pragmatic as a complement in clinical strategy making.

## CONFLICT OF INTEREST

The authors declare that they have no competing interests.

## ETHICS APPROVAL AND CONSENT TO PARTICIPATE

This retrospective study was approved by the Medical Ethics Committee of Xiangya Hospital, Central South University with approval no. 2018121147. Patients' informed consent was waived because of the retrospective nature of the study design.
