# Supplementary material for: Predicting pathological complete response by comparing MRI‐based radiomics pre‐ and postneoadjuvant radiotherapy for locally advanced rectal cancer
Source: Cancer Med. 2019 Oct 22;8(17):7244–52. doi: 10.1002/cam4.2636 (PMC6885895; doi:10.1002/cam4.2636)
Supplement: Supplementary file 1 [file CAM4-8-7244-s001.docx]

Supplementary material

**I : Definition of radiomics features**

We evaluated a total number of 161 MRI radiomics features, all radiomics features were calculated automatically with Imaging Biomarker Explorer Software(IBEX). The 161 features were divided in 7 groups as follows: (1) Shape, (2)Gray Level Cooccurrence Matrix-3D(GLCM-3D), (3)Gray Level Run Length Matrix(GLRLM), (4) Neighborhood Intensity Difference Matrix-3D(NIDM-3D). (5) Intensity Direct(ID), (6) Intensity Histogram(IH), (7) Gradient Orientation Histogram(GOH).

Group 1. Shape[[1](#_ENREF_1), [2](#_ENREF_2)]

1.Compactness 1 = (Volume)/(sqrt(pi)*(SurfaceArea)^(2/3)

2.Compactness 2 = 36*pi*(Volume^2)/((SurfaceArea)^3)

3.Convex : Measure the proportion of the pixels in the convex hull that are also in the region.

4.Convex Hull Volume: The mean volume of the 2D convex hulls that are the convex envelopes of each slice's binary mask.

5.Convex Hull Volume 3D: 3D volume of the convex hull that is the convex envelope of binary mask.

6.Max 3D Diameter: largest pairwise Euclidean distance between voxels on the surface of the tumor volume.

7.Mean Breadth: Denotes integral of mean curvature

8.Number Of Voxel : The number of voxels treating the edge voxels differently.

9.Orientation: Measures the angle between the x-axis and the major axis of the ellipse in 2D.

10.Roundness: Measures how much the binary mask is close to circle in 2D.

11.Spherical Disproportion 　**$spherical disproportion=\frac{A}{4\pi R^{2}}$**

12.Sphericity 　**$sphericity=\frac{\pi^{\frac{1}{3}}(6V)^{\frac{2}{3}}}{A}$**

13.Surface Area:The surface area of the binary mask. **$A=\sum_{i=1}^{N} \frac{1}{2}|a_{i}b_{i}\times a_{i}c_{i}|$**

14.Surface Area Density = (surface area of the binary mask)/(volume of the binary mask).

15.Volume:The physical volume treating the edge voxels differently.

16. Voxel Size: an important component of image quality. Voxel is the 3-D analog of a pixel. Voxel size is related to both the pixel size and slice thickness.

Group 2.Gray Level Cooccurrence Matrix-3D(GLCM-3D)[[1](#_ENREF_1), [3-5](#_ENREF_3)]

A GLCM is defined as $p (i, j, \delta, \alpha)$, a matrix with size $N_{g}\times N_{g}$ describing the second order joint probability function of an image, where the $(i, j)$th element represents the number of times the combination of intensity levels $i$ and $j$ occur in two pixels in the image, that are separated by a distance of $\delta$ pixels in direction $\alpha$, and $N_{g}$ is the number of discrete gray level intensities. In our study, distance $\delta$ was set to 1 and direction $\alpha$ to each of the 13 directions in three-dimensions.

Each 3D gray level co-occurrence based feature was calculated as the mean of the feature calculations for each of the 13 directions.

Let:

$P (i, j)$ be the co-occurrence matrix for an arbitrary $\delta$ and $\alpha$,

$N_{g}$be the number of discrete intensity levels in the image,

$\mu$ be the mean of $P \left( i, j \right)$,

$p_{x}\left( i \right)=\sum_{j=1}^{N_{g}} P (i, j)$ be the marginal row probabilities,

$p_{y}\left( i \right)=\sum_{i=1}^{N_{g}} P (i, j)$ be the marginal column probabilities,

$\mu_{x}$ be the mean of $p_{x}$,

$\mu_{y}$ be the mean of $p_{y}$,

$\sigma_{x}$ be the standard deviation of $p_{x}$,

$\sigma_{y}$ be the standard deviation of $p_{y}$,

$p_{x+y}\left( k \right)=\sum_{i=1}^{N_{g}} \sum_{j=1}^{N_{g}} P (i, j),i+j=k,k=2,3\ldots,2N_{g}$,

$p_{x-y}\left( k \right)=\sum_{i=1}^{N_{g}} \sum_{j=1}^{N_{g}} P (i, j),\left| i-j \right|=k,k=0,1\ldots,N_{g}-1$,

$HX=-\sum_{i=1}^{N_{g}} p_{x}(i)\log_{2} [p_{x}\left( i \right)]$ be the entropy of $p_{x}$,

$HY=-\sum_{i=1}^{N_{g}} p_{y}(i)\log_{2} [p_{y}\left( i \right)]$ be the entropy of $p_{y}$,

$H=-\sum_{i=1}^{N_{g}} \sum_{j=1}^{N_{g}} P (i, j)\log_{2} [P(i,j)]$ be the entropy of$P (i, j)$,

$HXY1=-\sum_{i=1}^{N_{g}} \sum_{j=1}^{N_{g}} P \left( i, j \right)\log(p_{x}(i)p_{y}(j))$

$HXY2=-\sum_{i=1}^{N_{g}} \sum_{j=1}^{N_{g}} p_{x}(i)p_{y}(j)\log(p_{x}(i)p_{y}(j))$

17. Autocorrelation=$\sum_{i=1}^{N_{g}} \sum_{j=1}^{N_{g}} ijP (i, j)$

18. Cluster prominence=$\sum_{i=1}^{N_{g}} \sum_{j=1}^{N_{g}} {{[i+j-\mu}_{x}\left( i \right)-\mu_{y}(j)]}^{4}P (i, j)$

19.Cluster shade=$\sum_{i=1}^{N_{g}} \sum_{j=1}^{N_{g}} {{[i+j-\mu}_{x}\left( i \right)-\mu_{y}(j)]}^{3}P (i, j)$

20. Cluster tendency=$\sum_{i=1}^{N_{g}} \sum_{j=1}^{N_{g}} {{[i+j-\mu}_{x}\left( i \right)-\mu_{y}(j)]}^{2}P (i, j)$

21. Correlation=$\frac{\sum_{i=1}^{N_{g}} \sum_{j=1}^{N_{g}} ijP \left( i, j \right)-\mu_{i}(i)\mu_{j}(j)}{\sigma_{x}(i)\sigma_{y}(j)}$

22. Contrast=$\sum_{i=1}^{N_{g}} \sum_{j=1}^{N_{g}} {|i-j|}^{2}P (i, j)$

23. Difference entropy=$\sum_{i=0}^{N_{g}-1} P_{x-y}(i)\log_{2} [P_{x-Y}\left( i \right)]$

24. Dissimilarity=$\sum_{i=1}^{N_{g}} \sum_{j=1}^{N_{g}} |i-j|P (i, j)$

25. GLCM Energy=$\sum_{i=1}^{N_{g}} \sum_{j=1}^{N_{g}} {[P\left( i,j \right)]}^{2}$

26.GLCM Entropy=**$-\sum_{i,j}^{Formula} g(i,j)\log_{2}(i,j)$**

27. Homogeneity1=$\sum_{i=1}^{N_{g}} \sum_{j=1}^{N_{g}} \frac{P(i,j)}{1+|i-j|}$

28. Homogeneity2=$\sum_{i=1}^{N_{g}} \sum_{j=1}^{N_{g}} \frac{P(i,j)}{1+{|i-j|}^{2}}$

29. Informational measure of correlation 1(IMC1)=$\frac{HXY-HXY1}{max\{HX,HY\}}$

30. Informational measure of correlation 2(IMC2)=$\sqrt{1-e^{-2(HXY2-HXY)}}$

31. Inverse difference moment normalized(IDMN)=$\sum_{i=1}^{N_{g}} \sum_{j=1}^{N_{g}} \frac{P(i,j)}{1+\frac{{|i-j|}^{2}}{N^{2}}}$

32. Inverse difference normalized(IDN)=$\sum_{i=1}^{N_{g}} \sum_{j=1}^{N_{g}} \frac{P(i,j)}{1+\frac{|i-j|}{N}}$

33. Inverse variance=$\sum_{i=1}^{N_{g}} \sum_{j=1}^{N_{g}} \frac{P(i,j)}{{|i-j|}^{2}},i\neq j$,

34. Maximum probability=max$\{ P (i, j)\}$

35. Sum average=$\sum_{i=2}^{2N_{g}} [iP_{x+y}(i)]$

36. Sum entropy=$-\sum_{i=2}^{2N_{g}} P_{x+y}(i)\log_{2} {[P}_{x+y}(i)]$

37. Sum variance=$\sum_{i=2}^{{2N}_{g}} \left( i-SE \right)^{2}P_{x+y}(i)$


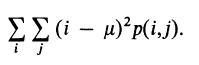
38.Variance=

Group 3. Gray Level Run Length Matrix[[6](#_ENREF_6)]

Run-Length metrics quantify gray level runs in an image. A gray level run is defined as the length in numbers of pixels, of consecutive that have the same gray level value. In a gray level run-length matrix $p (i, j,\theta)$, the $\left( i, j \right)$th element describes the number of times $j$a gray level $i$ appears consecutively in the direction specified by $\theta,$ and $N_{g}$ is the number of discrete gray level intensities.

Let:

$p \left( i, j,\theta\right)$ be the $\left( i, j \right)$th entry in the given run-length matrix $p$ for a direction $\theta$,

$N_{g}$ the number of discrete intensity values in the image,

$N_{r}$ the number of different run lengths,

$N_{p}$ the number of voxels in the mage,

39. Short run emphasis(SRE)=$\frac{\sum_{i=1}^{N_{g}} \sum_{j=1}^{N_{r}} [\frac{p \left( i, j,\theta\right)}{j^{2}}]}{\sum_{i=1}^{N_{g}} \sum_{j=1}^{N_{r}} [p \left( i, j,\theta\right)]}$

40. Long run emphasis(LRE)=$\frac{\sum_{i=1}^{N_{g}} \sum_{j=1}^{N_{r}} j^{2}p \left( i, j,\theta\right)}{\sum_{i=1}^{N_{g}} \sum_{j=1}^{N_{r}} p \left( i, j,\theta\right)}$

41. Gray level non-uniformity(GLN)=$\frac{\sum_{i=1}^{N_{g}} {[\sum_{j=1}^{N_{r}} p \left( i, j,\theta\right)]}^{2}}{\sum_{i=1}^{N_{g}} \sum_{j=1}^{N_{r}} p \left( i, j,\theta\right)}$

42: Run length non-uniformity(RLN)=$\frac{\sum_{j=1}^{N_{r}} {[\sum_{i=1}^{N_{g}} p \left( i, j,\theta\right)]}^{2}}{\sum_{i=1}^{N_{g}} \sum_{j=1}^{N_{r}} p \left( i, j,\theta\right)}$

43. Run percentage(RP)=$\sum_{i=1}^{N_{g}} \sum_{j=1}^{N_{r}} \frac{p \left( i, j,\theta\right)}{N_{p}}$

44. Low gray level run emphasis(LGLRE)=$\frac{\sum_{i=1}^{N_{g}} \sum_{j=1}^{N_{r}} [\frac{p \left( i, j,\theta\right)}{i^{2}}]}{\sum_{i=1}^{N_{g}} \sum_{j=1}^{N_{r}} [p \left( i, j,\theta\right)]}$

45. High gray level run emphasis(HGLRE)=$\frac{\sum_{i=1}^{N_{g}} \sum_{j=1}^{N_{r}} i^{2}p \left( i, j,\theta\right)}{\sum_{i=1}^{N_{g}} \sum_{j=1}^{N_{r}} p \left( i, j,\theta\right)}$

46. Short run low gray level emphasis(SRLGLE)=$\frac{\sum_{i=1}^{N_{g}} \sum_{j=1}^{N_{r}} [\frac{p \left( i, j,\theta\right)}{i^{2}j^{2}}]}{\sum_{i=1}^{N_{g}} \sum_{j=1}^{N_{r}} [p \left( i, j,\theta\right)]}$

47. Short run high gray level emphasis(SRHGLE)=$\frac{\sum_{i=1}^{N_{g}} \sum_{j=1}^{N_{r}} [\frac{p \left( i, j,\theta\right)i^{2}}{j^{2}}]}{\sum_{i=1}^{N_{g}} \sum_{j=1}^{N_{r}} [p \left( i, j,\theta\right)]}$

48. Long run low gray level emphasis(LRLGLE)=$\frac{\sum_{i=1}^{N_{g}} \sum_{j=1}^{N_{r}} [\frac{p \left( i, j,\theta\right)j^{2}}{i^{2}}]}{\sum_{i=1}^{N_{g}} \sum_{j=1}^{N_{r}} [p \left( i, j,\theta\right)]}$

49. Long run high gray level emphasis(LRHGLE)=$\frac{\sum_{i=1}^{N_{g}} \sum_{j=1}^{N_{r}} i^{2}j^{2}p \left( i, j,\theta\right)}{\sum_{i=1}^{N_{g}} \sum_{j=1}^{N_{r}} p \left( i, j,\theta\right)}$

Group 4.Neighborhood Intensity Difference Matrix-3D(NIDM-3D)[[7](#_ENREF_7)]

50.Coarseness


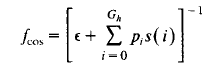


51.Contrast


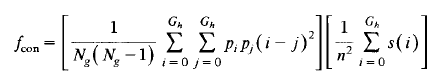


52.Busyness


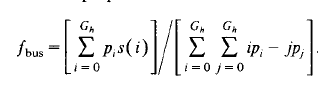


53.Complexity


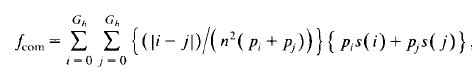


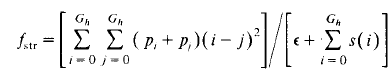
54.Texture Strength

Group 5.Intensity Direct(ID)[[1](#_ENREF_1)]

55.Energy=$\sum_{i}^{N} X{(i)}^{2}$

56.Energy Norm

57.Global Entropy: The intensity entropy among all the voxels.

58.Global Max:The intensity maximum among all the voxels.

59.Global Mean:The intensity mean among all the voxels.

60.Global Median:The intensity median among all the voxels.

61.Global Min:The intensity minimum among all the voxels.

62.Global Std:The intensity standard deviation among all the voxels.

63.Global Uniformity:The intensity uniformity among all the voxels.

64.Inter-Quartile Range:The interquartile range of the intensity values among all the voxels.

65.Kurtosis:Measure the peakedness of all the voxels' intensity.

66.Local Entropy Max:First, at each voxel, compute entropy in its neighborhood region. Then, compute the maximum among all the voxel's entropy calculated from step 1.

67.Local Entropy Mean:First, at each voxel, compute entropy in its neighborhood region. Then, compute the mean among all the voxel's entropy calculated from step 1.

68.Local Entropy Median:First, at each voxel, compute entropy in its neighborhood region. Then, compute the median among all the voxel's entropy calculated from step 1.

69.Local Entropy Min:First, at each voxel, compute entropy in its neighborhood region. Then, compute the minimum among all the voxel's entropy calculated from step 1.

70.Local Entropy Std:First, at each voxel, compute entropy in its neighborhood region. Then, compute the standard deviation among all the voxel's entropy calculated from step 1.

71.Local Range Max:First, at each voxel, compute range value (Max Value-Min Value) in its neighborhood region. Then, compute the median among all the voxel's range value calculated from step 1.

72.Local Range Mean:First, at each voxel, compute range value (Max Value-Min Value) in its neighborhood region. Then, compute the mean among all the voxel's range value calculated from step 1.

73.Local Range Median:First, at each voxel, compute range value (Max Value-Min Value) in its neighborhood region. Then, compute the median among all the voxel's range value calculated from step 1.

74.Local Range Min:First, at each voxel, compute range value (Max Value-Min Value) in its neighborhood region. Then, compute the minimum among all the voxel's range value calculated from step 1.

75.Local Range Std:First, at each voxel, compute range value (Max Value-Min Value) in its neighborhood region. Then, compute the standard deviation among all the voxel's range value calculated from step 1.

76.Local Std Max:First, at each voxel, compute standard deviation in its neighborhood region. Then, compute the maximum among all the voxel's standard deviation value calculated from step 1.

77.Local Std Mean:First, at each voxel, compute standard deviation in its neighborhood region. Then, compute the mean among all the voxel's standard deviation value calculated from step 1.

78.Local Std Median:First, at each voxel, compute standard deviation in its neighborhood region. Then, compute the median among all the voxel's standard deviation value calculated from step 1.

79.Local Std Min:First, at each voxel, compute standard deviation in its neighborhood region. Then, compute the minimum among all the voxel's standard deviation value calculated from step 1.

80.Local Std Std:First, at each voxel, compute standard deviation in its neighborhood region. Then, compute the standard deviation all the voxel's standard deviation value calculated from step 1.

81.Mean Absolute Deviation:The mean absolute deviation of the intensity values among all the voxels.

82.Median Absolute Deviation:The median absolute deviation of the intensity values among all the voxels.

83-101.Percentile:Percentiles of the intensity values among all the voxels. There were 19 percentiles from Percentile5 to Percentile95 with the interval of 5.

102-106.Quantile:Quantiles of the intensity values among all the voxels. Here, we have 5 quantiles, including Quantile0.025, Quantile0.25, Quantile0.5, Quantile0.75, Quantile0.975.

107.Range:The intensity range (Max Value-Min Value) among all the voxels.

108.Root mean square=$\sqrt{\frac{\sum_{i}^{N} X\left( i \right)^{2}}{N}}$

109.Skewness: Measure the asymmetry of all the voxels' intensity.

110. Variance=$\frac{1}{N-1}\sum_{i=1}^{N} \left( X\left( i \right)-\bar{X} \right)^{2}$

Group 6. Intensity Histogram[[1](#_ENREF_1)]

111.Inter-Quartile Range:The interquartile range of the occurrence probability values in the histogram.

112.Kurtosis:Measure the peakedness of the occurrence probability values in the histogram.

113.Mean Absolute Deviation:The mean absolute deviation of the occurrence probability values in the histogram.

114.Median Absolute Deviation:The median absolute deviation of the occurrence probability values in the histogram.

115-133.Percentile :Percentiles of the occurrence probability values in the histogram. There were 19 percentiles from Percentile5 to Percentile95 with the interval of 5.

134-152.Percentile Area:Percentiles of values in the accumulative histogram. There were 19 percentile Areas from PercentileArea5 to PercentileArea95 with the interval of 5.

153-157.Quantile:Quantiles of the occurrence probability values in the histogram. Here, we have 5 quantiles, including Quantile0.025, Quantile0.25, Quantile0.5, Quantile0.75, Quantile0.975.

158.Range:Measures the range (Max Value-Min Value) of the occurrence probability values in the histogram.

159.Skewness:Measure the asymmetry of the occurrence probability values in the histogram.

Group 7.Gradient Orientation Histogram(GOH)[[8](#_ENREF_8)]

160.Inter-Quartile Range:The interquartile range of the occurrence probability values in the histogram.

161.Kurtosis:Measure the peakedness of the occurrence probability values in the histogram.

162.Mean Absolute Deviation:The mean absolute deviation of the occurrence probability values in the histogram.

163.Median Absolute Deviation:The median absolute deviation of the occurrence probability values in the histogram.

164.Range:Measures the range (Max Value-Min Value) of the occurrence probability values in the histogram.

165.Skewness:Measure the asymmetry of the occurrence probability values in the histogram.

Rad-score = – 1.707223965

+ (3.025922752 * GOH-Skewness)

+ (2.555072335 * GLRLM-Run Length Non-uniformity)

+ (0.082640557 * ID-Local Entropy Max)

+ (0.033768506 * ID- Local Range Min)

+ (2850.029899 * NIDM-Coarseness)

–(0.326002518* Max 3D diameter)

+( 0.098407946* Surface Area Density)

Probability of pCR = e^x^ / (1 + e^x^), where e is the base of natural logarithms.

1. **Programing of Lasso logistic analysis in R**

library("glmnet")

df <- dataset #this is the required dataset and the first column should be the dependent variable.

x <- as.matrix(df [,-1])

y <- as.factor(df [,1])

cvfit <- cv.glmnet(x,y,family= "binomial")

plot(cvfit)

coef_n <- coef(cvfit, s = "lambda.1se")

coef_n

glmmod <- glmnet(x,y, alpha = 1,family = "binomial")

plot (glmmod, xvar = "lambda")

abline (v = log(cvfit$lambda.1se), lty = 2, lwd = 1)

**References**

1. Aerts, H.J., et al., *Decoding tumour phenotype by noninvasive imaging using a quantitative radiomics approach.* Nat Commun, 2014. **5**: p. 4006.

2. *Legland, D., Kiêu, K. & Devaux, M.-F. COMPUTATION OF MINKOWSKI MEASURES ON 2D AND 3D BINARY IMAGES. 2011 26, 10, doi:10.5566/ias.v26.p83-92 (2011).*

3. Leen-Kiat, S. and C. Tsatsoulis, *Texture representation of SAR sea ice imagery using multi-displacement co-occurrence matrices.* 1996. **1**: p. 112-114.

4. *Haralick, R. M., Shanmugam, K. & Dinstein, I. Textural Features for Image Classification. IEEE Transactions on Systems, Man, and Cybernetics SMC-3, 610-621, doi:10.1109/TSMC.1973.4309314 (1973).*

5. *Haralick, R. M. & Shapiro, L. G. Computer and Robot Vision. (Addison-Wesley Longman Publishing Co., Inc., 1992).*

6. *Xiaoou, T. Texture information in run-length matrices. IEEE Transactions on Image Processing 7, 1602-1609, doi:10.1109/83.725367 (1998).*

7. *Amadasun, M. & King, R. Texural Features Corresponding to Texural Properties. IEEE Transactions on Systems, Man and Cybernetics 19, 1264-1274, doi:10.1109/21.44046 (1989).*

8. Pallavi, T., et al., *Texture Descriptors to distinguish Radiation Necrosis from Recurrent Brain Tumors on multi-parametric MRI.* Proc SPIE Int Soc Opt Eng, 2014. **9035**: p. 90352B.
